# A Telomerase-Derived Peptide Exerts an Anti-Hepatitis B Virus Effect *via* Mitochondrial DNA Stress-Dependent Type I Interferon Production

**DOI:** 10.3389/fimmu.2020.00652

**Published:** 2020-05-21

**Authors:** Yu-Min Choi, Hong Kim, Seoung-Ae Lee, So-Young Lee, Bum-Joon Kim

**Affiliations:** Department of Microbiology and Immunology, Biomedical Sciences, Liver Research Institute and Cancer Research Institute, College of Medicine, Seoul National University, Seoul, South Korea

**Keywords:** covalently closed circular DNA, heme oxygenase 1, mitochondrial ROS, phagosomal escape, type I interferons

## Abstract

Previously, a telomerase-derived 16-mer peptide, GV1001, developed as an anticancer vaccine, was reported to exert antiviral effects on human immunodeficiency virus or hepatitis C virus in a heat shock protein-dependent manner. Here we investigated whether GV1001 exerts antiviral effects on hepatitis B virus (HBV) and elucidated its underlying mechanisms. GV1001 inhibited HBV replication and hepatitis B surface antigen (HBsAg) secretion in a dose-dependent manner, showing synergistic antiviral effects with nucleos(t)ide analogs (NAs) including entecavir and lamivudine. This peptide also inhibited viral cccDNA and pgRNA. The intravenous GV1001 treatment of transgenic mice had anti-HBV effects. Our mechanistic studies revealed that GV1001 suppresses HBV replication by inhibiting capsid formation *via* type I interferon-mediated induction of heme oxygenase-1 (HO-1). GV1001 promoted the mitochondrial DNA stress-mediated release of oxidized DNA into the cytosol, resulting in IFN-I-dependent anti-HBV effects *via* the STING-IRF3 axis. We found that the anti-HBV effect of GV1001 was due to its ability to penetrate into the cytosol *via* extracellular heat shock protein, leading to phagosomal escape-mediated mtDNA stress. We demonstrated that the cell-penetrating and cytosolic localization capacity of GV1001 results in antiviral effects on HBV infections *via* mtDNA stress-mediated IFN-I production. Thus, GV1001, a peptide proven to be safe for human use, may be an anti-HBV drug that can be synergistically used with nucleot(s)ide analog.

## Graphical Abstract

Schematic representation showing the mechanisms regarding the anti-HBV effect of GV1001. GV1001 exerted mtDNA stress-mediated IFN-I production *via* STING-dependent oxidized cytosolic DNA sensing, which was mediated by its eHSP-dependent cytosolic access and phagosomal escape in hepatocytes. The enhanced IFN-I production by GV1001 exerted an anti-HBV effect *via* interfering with stable capsid formations by enhanced HO-1 expression.

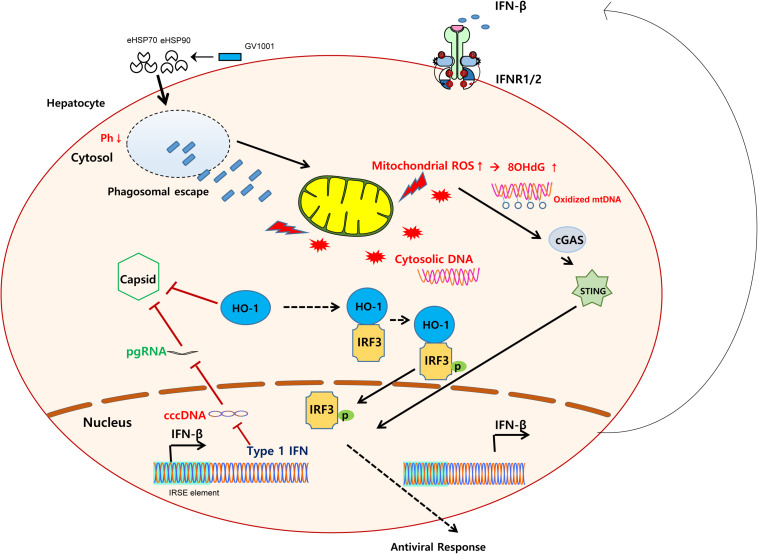

## Introduction

Hepatitis B virus infection is associated with adverse outcomes of liver diseases, including cirrhosis, hepatic decompensation, and hepatocellular carcinoma (HCC). The annual number of deaths caused by HBV-related diseases is approximately 887,000 worldwide (1). Although there is variation according to geography, endemicity, and viral genotypes or the prevalence of vertical transmission, approximately 12–20% of the infected patients will have a 5-year progression from CHB to liver cirrhosis (LC), and the 5-year cumulative risk of HCC progression is estimated to be between 10 and 17% in LC patients (2).

Unfortunately, despite their high efficacies, all currently approved HBV life cycle inhibitors, including two exogenous interferon (IFN)-based therapies—IFN and pegylated IFN—and five oral nucleot(s)ide analogs (NAs)—lamivudine (LMV), adefovir dipivoxil, entecavir (ETV), telbivudine, and tenofovir disoproxil fumarate, have their own limitations. Long-term NA treatment results in NA-resistant viral strains and cannot completely eradicate HBV cccDNAs in infected hepatocytes (3).

Exogenous IFN-related treatments can eliminate HBV cccDNA in infected hepatocytes *via* epigenetic regulation, which could lead to HBsAg seroconversion in chronic patients, a signature of complete remission. However, these treatments are associated with a high incidence of adverse effects (4). Therefore, novel anti-HBV agents with improved efficacy and safety are urgently needed.

Mitochondria are central eukaryotic organelles of energy production, which maintain mitochondrial DNA (mtDNA) encoding essential protein subunits involved in driving mitochondrial respiration and ATP production (5). In addition to energy production, mitochondria are involved in other cellular functions, including anabolic and catabolic pathways, apoptosis regulation, calcium homeostasis, and reactive oxygen stress (ROS) signaling (6, 7). Moreover, mitochondria were shown to trigger innate immune responses *via* the release of damage-associated molecular patterns (DAMPs), such as oxidized mtDNA during cellular stress, infections, or injury (8). Cytosolic mtDNA has antiviral activity against various viral infections, including HBV, hepatitis C virus (HCV), and HSV, *via* production of innate cytokines such as type I interferon (IFN-I) or IL-1β (9, 10). Therefore, agents that induce mtDNA stress have therapeutic potential as antiviral drugs for HBV infections.

GV1001, a human telomerase reverse transcriptase-derived 16-amino-acid peptide, was designed as an anticancer vaccine for several cancers, including advanced pancreatic cancer, non-small cell lung cancer, and melanoma (11–13). In addition to its anticancer effects, GV1001 has various biological activities including anti-inflammatory (14), anticancer (15), anti-apoptotic, and antioxidant roles (16). Furthermore, we recently reported that GV1001 has antiviral effects against HCV and human immunodeficiency virus type 1 (HIV-1) *via* extracellular heat shock protein (eHSP)-GV1001 binding-mediated cell signaling (17, 18). Therefore, we aimed to explore the possible antiviral role of GV1001, a safe drug in human, in HBV infections and to elucidate its underlying mechanism against HBV infections, mainly focusing on mtDNA stress-mediated IFN-I production.

## Materials and Methods

### Cells and Reagents

HepG2 cells were maintained in Eagle’s minimum essential medium (MEM) containing 10% fetal bovine serum (FBS), penicillin/streptomycin (PS) (100 U/ml), and N-2-hydroxyethylpiperazine-N-2-ethane sulfonic acid (25 mM). HepG2-2.15 cells were cultured in Dulbecco’s modified Eagle’s medium (DMEM) containing 10% FBS, and PS (100 U/ml). Huh-7 cells were maintained in RPMI 1640 medium containing 10% FBS and PS (100 U/ml). Antibodies against HSP90 (sc-101494), HSP70 (sc-32239), heme oxygenase 1 (sc-10789), GAPDH (sc-25778 and sc-293335), HBsAg (sc-52410), pSTAT1 (sc-7988), LAMP-1 (sc-20011 and sc-17768), heme oxygenase 1 siRNA (sc-35554), and control siRNA (sc-30007) were purchased from Santa Cruz Biotechnology (Santa Cruz, Dallas, TX, United States). Antibodies against IRF3 (#4962), pIRF3 (#4947S), STAT1 (#9172), and LC3B (#2775S) were purchased from Cell Signaling Technology, Inc. (CST). Antibodies against HBV core protein were purchased from Dako (B0586) and Abcam (ab18686). Bafilomycin-A1 (B 1793) was obtained from Sigma-Aldrich (St. Louis, MO, United States). A luciferase assay kit (E1501) was purchased from Promega, and the MitoSOX Red mitochondrial superoxide indicator (M36008) was purchased from Invitrogen.

### *In vivo* Assay and Hydrodynamic Injection

Transgenic (TG) mice were generated by transferring the pHY92-1.1x-HBV-full genome plasmid (genotype A2) into C57B1/6N mice, and the TG mice used in this study constitutively express the HBV genome with the W4P mutation in the preS1 region (19). The age-matched TG mice were injected with GV1001 (50 μg/kg) and LMV (500 μg/kg) twice per week. Serum was collected from the orbital sinus of the mice at 4 and at 8 weeks. All animal experiments were approved by the Institutional Animal Care and Use Committee of the Seoul National University College of Medicine (SNU-170308).

### Anti-viral Effect Assay

For the analysis of the anti-HBV effect of GV1001, HepG2, Huh-7, or HepG2-2.15 cells were treated with PBS, entecavir, lamivudine, or GV1001 and incubated for 24 or 48 h. The supernatants were collected, and HBsAg and HBeAg ELISAs were conducted using a commercial Bioelisa HBsAg color ELISA Kit (BIOKIT, Barcelona, Spain) and a HBeAg ELISA kit (AccuDiag^TM^, DIAGNOSTIC AUTOMATION, INC., Woodland Hills, CA, United States), respectively, according to the manufacturer’s procedures. Cell pellets were harvested and subjected to RNA extraction for RT-qPCR to determine the viral amount.

### HBV Viral Quantification and mRNA

For evaluating the HBV viral titers, HBV genomes from cell culture supernatants and pellets were purified using the QIAamp Blood DNA extraction kit (QIAGEN, Hilden, Germany) and quantitated by qPCR using a primer pair specific to the small S gene (SF: 5′-TTG ACA AGA ATC CTC ACA ATA CC-3′) and antisense primer SR (positions 309–328, 5′-GGA GGT TGG GGA CTG CGA AT-3′). The HBV DNA plasma standard containing 1 × 10^6^ HBV genomic DNA copies/ml (HBV DNA Quantiplex, Chiron) was used to standardize the viral titers. The following primer sets were used to investigate the mRNA expression levels with RT-qPCR: 18S-F: 5′-AGTCCCTGCCCTTTGTACACA-3′ and 18S-R: 5′-CGATCCGAGGGCCTCACTA-3′, HO-1-F: 5′-TTG CCAGTGCCACCAAGTTC-3′, and HO-1-R: 5′-TCAGCAG CTCCTGCAACTCC-3′, IFNb-F: 5′-TTGTGCTTCTCCACTA CAGC-3′ and hIFNb-R: 5′-CTGTAAGTCTGTTAATGAAG-3′, mtDNA1-F: 5′CATGCCCATCGTCCTAGAAT-3′ and mtDNA1-R: 5′-ACGGGCCCTATTTCAAAGAT-3′, mtDNA2-F: 5′-CCCTAACACCAGCCTAACCA-3′ and mtDNA2-R: 5′-AA AGTGCATACCGCC7AAAAG-3′, mtDNA3-F: 5′-TCCAACT CATGAGACCCACA-3′ and mtDNA3-R: 5′-TGAGGCT TGGATTAGCGTTT-3′).

### HBV pgRNA Assay

Total RNA from cell pellets was extracted using TRIzol reagent (Invitrogen, Carlsbad, CA, United States) according to the provided protocol. The RNA samples were incubated with RQ1 DNase (Promega, United Kingdom) for 60 min at 37°C, mixed with 1 μl of stop solution, incubated at 65°C for 10 min to inactivate the DNase, and stored at −80°C until use. For detection of viral pgRNA, 2 μg of RNA was reverse-transcribed and amplified by the Reverse Transcription System (Promega, United Kingdom). Next, 2 μl of each cDNA was quantified by RT-qPCR analysis, and the 18S rRNA gene was used to normalize the RNA samples.

### HBV cccDNA Assay

HepG2 cells transiently transfected with a linearized 1.2x genotype C2 HBV plasmid were collected and incubated in lysis buffer A (10 mM Tris–HCl, pH 7.4, 1 mM EDTA, 50 mM NaCl, and 1% NP-40) for 10 min at 4°C. The lysates were centrifuged for 5 min at 14,000 rpm, and the nuclear pellet in lysis buffer B (10 mM Tris–HCL, 10 mM EDTA, 150 mM NaCl, 0.5% SDS) was sonicated at three to four pulses of 60% power and incubated overnight at 37°C after treatment with 0.5 mg/ml proteinase K. After the lysates were extracted with phenol–chloroform (1:1) and precipitated with ethanol, 1 μg DNA was treated with 10 U of PSAD (Plasmid safe DNase I, Epicenter, PA, United States) for 45 min at 37°C. The reaction was stopped by incubating for 30 min at 70°C. RT-qPCR was performed, and the 18S rRNA was used for normalization.

### siRNA Transfection of HepG2 and HepG2-2.15 Cells

HepG2 cells and HepG2-2.15 cells were grown in six-well plates to 70–80% confluency. siRNA transfection was carried out using Lipofectamine 3000 following the manufacturers’ procedures (Santa Cruz and Thermo Fisher Scientific). The cells were incubated with 1 ml of OPTI-MEM containing a mixture of siRNA (75 pmol) and Lipofectamine 3000 reagent (7.5 μl) for 6 h. The transfection solutions were replaced with MEM or DMEM containing 2% FBS in the presence of GV1001 or PBS. Then, the cells were incubated for 24 h, and the supernatants and pellets were collected for ELISAs and RT-qPCR assays.

### Cellular Uptake Mechanism

HepG2-2.15 cells were treated with anti-HSP70 (1 μg/ml), anti-HSP90 (1 μg/ml), or anti-GAPDH (1 μg/ml) antibodies for 1 h. After neutralization with HSP70, HSP90, or GAPDH antibodies, the cells were inoculated for 24 h in the presence of GV1001 or PBS. The role of the corresponding proteins in the uptake process was confirmed, and the effect on the virion level in the presence of GV1001 was measured by qPCR.

### Cell Cytotoxicity Assay

HepG2 and Huh-7 cells were seeded (1 × 10^4^ cells) in 96-well microplates and incubated with increasing concentrations of GV1001 for 3 days. Cell viability was determined using the MTT assay kit (Promega, Fitchburg, WI, United States). For analysis of the cytotoxicity of GV1001 and entecavir, CytoTox 96 Non-Radioactive Cytotoxicity Assays (G1780, Promega, Fitchburg, WI, United States) of the collected supernatant were performed according to the manufacturer’s protocol.

### Western Blot Analysis

The harvested cells were lysed using RIPA buffer (CST, #9806) containing protease inhibitor and phosphate inhibitor (Hoffmann-La Roche Inc.) and incubated for 20 min on ice. The lysed cells were centrifuged for 30 min at 13,000 rpm, and the lysates were collected for Western blotting. A Bradford assay was performed for protein quantification. Then, 5X loading buffer and PBS were added for protein quantification, and the samples were boiled for 5 min and chilled on ice. Protein samples were separated by electrophoresis, transferred to NC membranes, and blocked for 1 h with 5% skim milk or bovine serum albumin. The membranes were incubated overnight at 4°C with primary antibodies (1:1,000). On the next day, the membranes were washed with 0.1% Tween-20 in Tris–buffered saline and incubated in HRP secondary antibodies (1:2,000) for 2 h. After ECL solution was applied to the membrane, proteins were detected on an imager (LAS 2000).

### Confocal Microscopy

#### Confocal Microscopy With Fluorescein Isothiocyanate-Labeled Peptides and Bafilomycin A1

Cells were seeded and cultivated in two-chamber glass slides (Nunc, Roskilde, Denmark) for 12 h. After the cells were washed with PBS, they were incubated in serum-free OPTI-MEM for an hour. Fluorescein isothiocyanate (FITC)-labeled peptides were added to the cells for 2 h in the presence of Bafilomycin A1 or PBS. The cells were fixed with 4% paraformaldehyde solution for 10 min at room temperature (RT). After fixation, the cells were permeabilized with 0.1% Triton-X 100 for 10 min. Nuclear staining was performed with DAPI, and the cells were mounted in a mounting medium (VECTASHIELD Antifade Mounting Medium, H-1000).

#### Confocal Microscopy With MitoSOX

The cells were seeded and cultivated in two-chamber glass slides (Nunc, Roskilde, Denmark) for 2 h. After the cells were washed with PBS, they were incubated in DMEM containing 2% FBS in the presence of GV1001 or PBS for 12 h. The cells were washed with PBS and stained with MitoSOX (1 μM) for 10 min. The cells were fixed with 4% paraformaldehyde solution for 10 min at room temperature. After fixation, the cells were permeabilized with 0.1% Triton-X 100 for 10 min. Nuclear staining was performed with DAPI, and the cells were mounted in a mounting medium (VECTASHIELD Antifade Mounting Medium, H-1000). Images were captured using a100× oil immersion objective lens.

### Flow Cytometry With MitoSOX

To determine the mitochondrial superoxide level by flow cytometry, we treated the cells with GV1001 or PBS for either 6 or 12 h and stained them with 5 μM MitoSOX Red according to the manufacturer’s protocol. The measurements were carried out using a FACS Calibur system (BD Bioscience, San Jose, CA, United States).

### ELISAs for 8-OHdG

After the HepG2-2.15 cells were seeded into six-well plates for 12 h, they were treated with PBS (0.5%) or GV1001 (5 or 10 μM) for 12 h. From the pellets, genomic DNA was extracted using a QIAamp Blood DNA extraction kit (QIAGEN, Hilden, Germany). For the detection of 8-hydroxy-2′-deoxyguanosine (8-OHdG) activity, competitive ELISAs from an 8-OHdG analysis kit (OxiSelect Oxidative DNA Damage ELISA kit, Cell Biolabs, San Diego, CA, United States) was used according to the manufacturer’s protocol.

### Type I IFN Bioassay and Neutralization Assay

For the indirect measurement of IFN levels using luciferase reporter genes, hMH55-293-ISRE cells integrating IFN-sensitive response elements (ISREs) associated with the luciferase reporter gene at the 3′ end were established using 5 μg of puromycin (Sigma-Aldrich, St. Louis, MO, United States). After the supernatant was collected from the cells treated with each reagent for 24 or 48 h, it was added to the hMH55-293-ISRE cells for 6 h. After incubation, the cells were washed with PBS and lysed by Reporter Lysis Buffer (E1500, Promega, Fitchburg, WI, United States) for 30 min at RT. Then, the luciferase assay reagent (E1500, Promega, Fitchburg, WI, United States) was added, and the luminescence was measured using a TECAN m200 reader (TECAN, Switzerland). For the neutralization assay, the HepG2-2.15 cells were seeded into six-well plates for 12 h. On the next day, the cells were pre-incubated with anti-IFNAR2 and anti-GAPDH antibodies for 2 h at RT on the rotator. After the cells were washed with PBS, they were treated with PBS (0.5%) or GV1001 (5 or 10 μM) for 12 h.

### Statistical Analysis

Statistical comparisons between the control and tested groups were analyzed using one-way ANOVA. The *p*-value of statistical significance was set at *p* < 0.05 (^∗^), 0.01 (^∗∗^), or 0.001 (^∗∗∗^). All the experiments were independently repeated three times.

## Results

### Anti-HBV Effect of GV1001 on *in vitro* Hepatocyte Cultures

**FIGURE 1:**
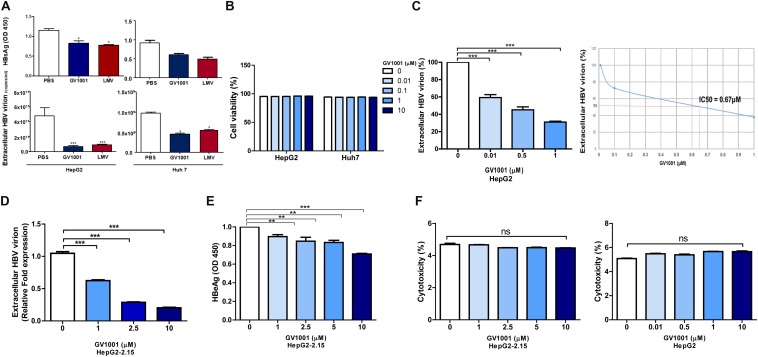
The anti-HBV effect of GV1001 on human hepatocytes. **(A)** HBsAg and extracellular HBV DNA levels were evaluated. PBS (0.5%), GV1001 (10 μM), and LMV (10 μM) were added to HepG2 cells and Huh-7 cells transiently transfected with the pHBV-1.2X-wild-type plasmid. **(B)** Cell viability assays (MTS) were conducted. **(C)** Quantitative PCR was performed on the supernatant of HepG2 cells transfected with 1.2x-WT plasmid and treated with GV1001 at different doses. The IC50 was calculated. **(D,E)** Extracellular HBV virion and HBeAg levels were measured using qPCR and ELISAs from the supernatants of HepG2-2.15 cells after the administration of phosphate-buffered saline and GV1001 for 48 h. **(F)** A lactate dehydrogenase assay was carried out on HepG2-2.15 and HepG2 cells to determine the cytotoxicity induced by GV1001. Data represent the mean ± SD of three independent experiments. *p < 0.05, **p < 0.01 and ***p < 0.001 versus PBS.

To determine the antiviral effect of GV1001 on HBV, we first examined its effect on hepatocyte, HepG2, and Huh-7 cells transiently transfected with a 1.2x genotype C2 HBV genome plasmid *via* the analysis of secreted virion and HBsAg levels. We observed an anti-HBV effect of GV1001 on both transfected cells in a dose-dependent manner 48 h after transfection with no cell cytotoxicity. After the treatment with PBS, LMV, or GV1001 for 2 days, the HBsAg and the extracellular virion DNA levels were measured. The GV1001-treated cells in both groups showed decreased HBsAg and extracellular HBV virion level compared with the PBS groups, and the LMV-treated group showed a similar effect to the GV1001-treated group (Figure 1A). No cytotoxicity was observed in either cell lines after the GV1001 treatment, which was confirmed by MTS assay (Figure 1B). Furthermore, extracellular viral DNA was significantly decreased by GV1001 in a dose-dependent manner in HepG2 cells. The mean IC50 of GV1001 on HepG2 cells, transfected with the 1.2x genotype C2 HBV genome plasmid, was approximately 0.67 μM (Figure 1C). Next, we evaluated the anti-HBV effect of GV1001 in stable HepG2-2.15 cell lines that constitutively expressed HBV virions. GV1001 reduced the extracellular HBV virion and HBeAg levels in HepG2-2.15 cells in a dose-dependent manner (Figures 1D,E), while the reduction in HBeAg level was not definite compared with the reduction level in extracellular HBV virion and in IC50 calculation. GV1001-induced cytotoxicity was not observed on both HepG2 and HepG2-2.15 cells (Figure 1F). Together our data indicated that GV1001 had an antiviral effect on HBV infection in a dose-dependent manner in hepatocyte cultures *in vitro*.

### Suppressive Effect on HBV cccDNA, pgRNA, and Nucleocapsid Formation by GV1001 in HepG2 Cells

**FIGURE 2:**
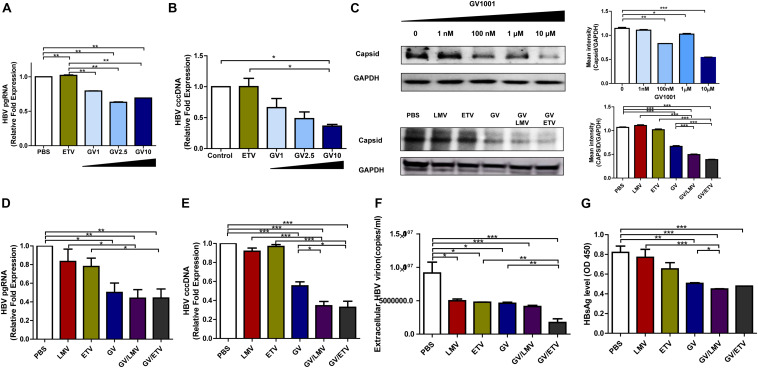
Suppressive effects of GV1001 on HBV cccDNA, pgRNA, and nucleocapsid and synergistic antiviral effects of GV1001 and Nucleos(t)ide analogs. **(A)** RNA was isolated from HepG2 cells transfected with pHBV-1.2x-WT and 3.5 kb/pgRNA and quantified using selective primers and probes. **(B)** cccDNA was extracted from the nuclei of HepG2 cells transiently transfected with pHBV-1.2X-WT after treatment with phosphate-buffered saline (PBS) (0.5%), entecavir (ETV) (30 nM), and GV1001 (10 μM). RT-qPCR was conducted using primers to detect cccDNA and human 18S primers to normalize the DNA samples. **(C)** After treatment with PBS and GV1001 (1 nM–10 μM), capsid formation of HepG2 transfected cells was detected by Western blots using native gels. After treatment with PBS, GV1001 (10 μM), lamivudine (LMV) (10 μM), and ETV (30 nM) or co-treatment (combination of each single dose), capsid formation of HepG2-2.15 stable cells was detected by Western blots using native gels. **(D–G)** Synergistic anti-HBV effect of GV1001 and NAs (LMV and ETV). HBV pgRNA **(D)** and cccDNA **(E)** were measured following treatment with each drug or co-treatment with GV1001 and NAs. **(F)** Quantitative qPCR was performed to detect viral titers with each compound or combination. **(G)** HBSAg ELISA was performed following treatment with each drug or co-treatment with GV1001 and NAs. Data represent the mean ± SD of three independent experiments. *p < 0.05, **p < 0.01 and ***p < 0.001.

The cccDNA, which can serve as a template for viral RNAs as an episome in the nucleus, is a major challenge in HBV therapy. Current NA-based treatment has rarely been reported to eliminate cccDNA or pgRNA in the nuclei of infected hepatocytes (20). Therefore, we examined whether GV1001 could inhibit HBV cccDNA or pgRNA in infected hepatocytes. To this end, we analyzed HepG2 cells transiently transfected by a linearized 1.2x genotype C2 HBV plasmid and assessed those replication capacity depending on the treatment of GV1001. The cccDNA and pgRNA transcript levels were significantly reduced by GV1001, but not ETV, compared with PBS (Figures 2A,B). Stable nucleocapsid formation could contribute to persistent HBV infections by promoting cccDNA production *via* its nuclear transport (21). To determine the inhibitory effect of GV1001 on HBV nucleocapsid formation, we conducted Western blotting for capsid detection on non-denatured gels using a cell pellet of HepG2 cells transiently transfected with the linearized 1.2x genotype C2 HBV plasmid. GV1001 treatment reduced the virion nucleocapsid levels in a dose-dependent manner (Figure 2C).

Next, we examined whether co-treatment with GV1001 and NAs had a synergistic antiviral effect. Co-treatment of GV1001 (10 μM) with LMV (10 μM) or ETV (10 μM) into HegG2.2.15 cells significantly reduced the pgRNA, cccDNA, extracellular virion DNA, HBsAg, and nucleocapsid levels compared with single-drug treatments (Figures 2C–G). Together, these results suggest that the anti-HBV mechanism induced by GV1001 may be attributed to the inhibition of cccDNA, pgRNA, and nucleocapsid formation *via* modulation of the host cell signaling involved in epigenetic modification, unlike NAs, which directly act on HBV polymerase.

### Anti-HBV Effect of GV1001 *in vivo*

**FIGURE 3:**
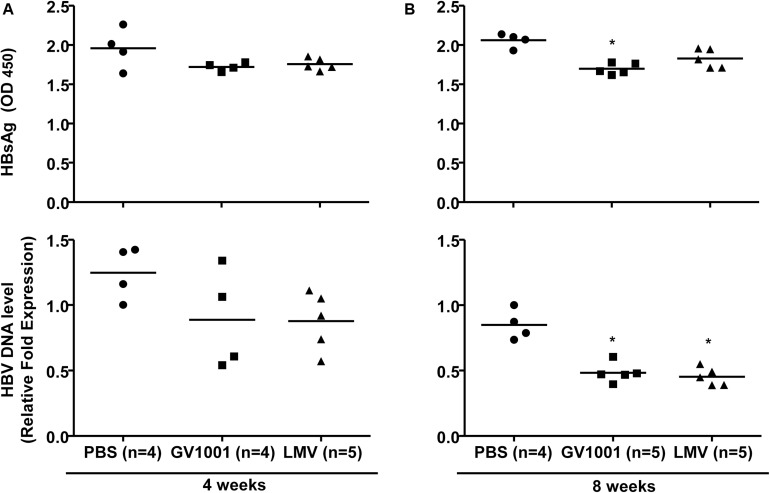
Anti-HBV effect of GV1001 on a transgenic mouse model. **(A)** Female transgenic mice were injected with phosphate-buffered saline, lamivudine, (0.5 mg/kg) or GV1001 (0.05 mg/kg), twice weekly. At 4 and at 8 weeks later, HBsAg from mouse serum was measured by ELISAs. **(B)** After the extraction of viral DNA from serum, HBV DNA was quantified by qPCR. This study was conducted in accordance with the guidelines established by the Seoul National University Institutional Animal Care and Use Committee (Approval No. SNU-111025-6-3). Data represent the mean ± SD of three independent experiments. *p < 0.05, **p < 0.01, and ***p < 0.001 versus PBS.

To examine the anti-viral effect of GV1001 in an *in vivo* mouse model, we intravenously injected GV1001 (50 μg/kg) or LMV (500 μg/kg) into a transgenic mouse model expressing HBV virions containing W4P mutation in the preS1 region (22) twice per week. The HBV DNA and HBsAg levels were measured from the mouse serum obtained *via* orbital sinus blood collection at 4 and 8 weeks. GV1001 did not significantly reduce the serum HBsAg or extracellular virion levels in mice after 4 weeks of infection compared with PBS, but a significant reduction in serum virions was found in GV1001-treated mice after 8 weeks of infection. The treatment of TG mice with GV1001 led to a mean HBsAg reduction of 20% compared to PBS, which had a limited effect on HBsAg level (Figures 3A,B). The LMV treatment had similar anti-HBV effects as GV1001 (Figure 3B). These results suggest that GV1001 also exerted antiviral effects on HBV infection in HBV transgenic mice.

### The Anti-HBV Effect of GV1001 Depends on IFN-I Production *via* a STING-IRF3 Axis

**FIGURE 4:**
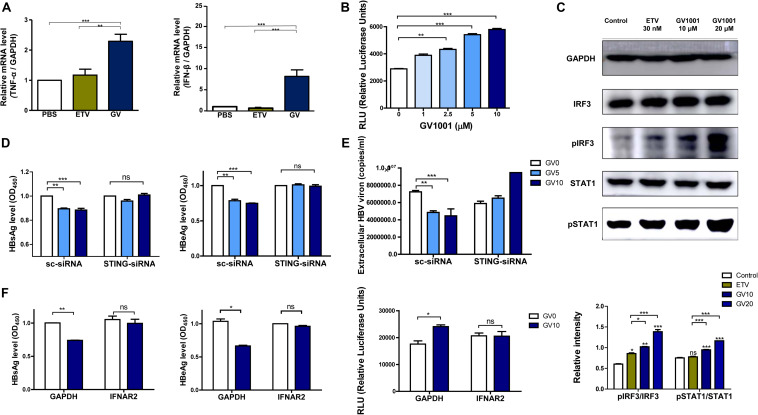
Anti-HBV effect of GV1001 depends on STING-mediated IFN-I signaling. **(A)** Total RNA was extracted from HepG2-2.15 cells treated with phosphate-buffered saline (PBS), entecavir (ETV) (30 nM), and GV1001 (10 μM) for 48 h, and the mRNA levels, hTNFα, and hIFNβ were evaluated *via* RT-qPCR. **(B)** The type 1 IFN expression level following the treatment of GV1001 was measured using hMH55-293-ISRE cells and a luciferase assay kit. **(C)** Western blot analysis of HepG2-2.15 cells treated with PBS, ETV, and GV1001 for 48 h was performed using GAPDH, IRF3, phospho-IRF3, STAT1, and phospho-STAT1 antibodies. **(D,E)** HepG2-2.15 cells were transfected with scramble- or STING-siRNA for 6 h and treated with GV1001 for 24 h. HBsAg, HBeAg, and extracellular HBV virion levels were measured using ELISAs and qPCR. **(F)** HepG2-2.15 cells were pre-incubated with GAPDH and IFNAR2 antibodies (2.5 μg/ml) for 2 h at room temperature and treated with PBS or GV1001 for 12 h. HBsAg, HBeAg, and type 1 IFN levels were measured. Data represent the mean ± SD of three independent experiments. *p < 0.05, **p < 0.01, and ***p < 0.001 versus PBS.

The above results showing the anti-HBV effect of GV1001 were due to the inhibition of cccDNA and HBV pgRNA, suggesting that the antiviral activity may be mediated *via* IFN-I production. To address this issue, we first examined whether GV1001 induced the transcription of IFN-I, which is responsible for the reduction of HBV cccDNA and nucleocapsid formation, using RT-qPCR. Our data showed that the gene transcriptions of IFN-β and TNF-α were significantly increased in GV1001-treated HepG2 cells transiently transfected with the HBV genome compared with PBS- or ETV-treated cells (Figure 4A), suggesting a positive role of IFN-I signaling in the anti-HBV effect of GV1001, which was distinct from the ETV-mediated anti-HBV effect. Then, we measured the secreted IFN-I level using HEK293-ISRE-Luc cells, which express luciferase luminescence according to the IFN level (23). The luminescence induced by GV1001 was significantly increased in a dose-dependent manner, confirming that GV1001 induces IFN-I production (Figure 4B). GV1001 also increased the phosphorylated IRF3 and phosphorylated STAT-1 levels, which are both considered as key regulatory factors inducing IFN-I (24), as confirmed by Western blot (Figure 4C), suggesting that the anti-HBV effect of GV1001 may be exerted *via* the IRF-3–IFN-I axis. There are two general upstream signaling pathways for IFN-I production related to HBV infections: the RIG-dependent pathway (25) and the cGAS–STING-dependent pathway (26). Because there was no difference in the antiviral effect of GV1001 between Huh-7 and Huh-7.5 cell lines defective in the RIG-I pathway (data not shown), we hypothesized that IFN-I production by GV1001 may be due to the cGAS–STING dependent pathway. First, we knocked down STING with siRNA to demonstrate the involvement of cGAS–STING signaling in the IFN-I production of GV1001. We found that the reduced HBsAg, HBeAg, and extracellular HBV virion levels mediated by GV1001 in scramble (sc) siRNA-transfected HepG2 cells were not observed or even increased in STING siRNA-transfected cells, suggesting that the anti-HBV effect depends on the STING-1-mediated signal pathway (Figures 4D,E). In addition, to further assess the IFN-I pathway dependence of the anti-HBV effect of GV1001, we assayed the anti-HBV effect of GV1001 after treatment with an IFN receptor-neutralizing antibody (IFNAR2). The anti-HBV effect of GV1001 observed in the GAPDH group was not found in the IFNAR2 group (Figure 4F). There was no difference in the IFN-I levels between the cells with and without GV1001 in the IFNAR2 group, suggesting that the anti-HBV effect of GV1001 depends on the IFN-I pathway. Taken together, these results demonstrated that GV1001 exerts anti-HBV effects *via* the stimulation of IFN-I production by the cGAS–Sting–IRF3 pathway.

### The Induction of IFN-I by GV1001 Is Dependent on the Release of Oxidized DNAs Into the Cytosol by Mitochondrial DNA Stress

**FIGURE 5:**
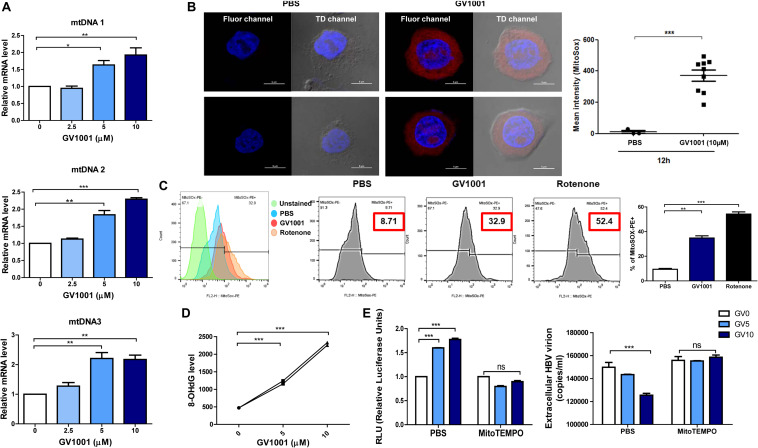
GV1001 leads to mtDNA stress-mediated IFN-I production in hepatocytes. **(A)** Cytosolic DNA was isolated from HepG2-2.15 cells treated with GV1001 for 48 h to detect cytosolic mtDNA. Quantitative cytosolic mtDNA levels were measured by RT-qPCR. **(B)** HepG2-2.15 cells were stained for mitochondrial superoxide (MitoSOX, red) and nuclei (DAPI, blue). The right panel of the confocal images shows the merged images in TD channel. **(C)** Mitochondrial ROS (mtROS) was stained with MitoSOX (5 μM) and assessed by flow cytometry following the treatment with 10 μM GV1001 for 12 h. Rotenone was used as a positive control for mtROS production. Fluorescence peak was presented and analyzed as the coefficient of variance (CV) and/or standard deviation (SD) of the arithmetic of the fluorescence intensity, and a statistical comparison of populations was performed. **(D)** An 8-OHdG ELISA was performed with genomic DNA extracted from HepG2-2.15 cells treated with phosphate-buffered saline or GV1001 (5 or 10 μM) for 12 h. **(E)** The relationship between mitochondrial stress and the type 1 IFN-mediated anti-HBV effect of GV1001 was confirmed using MitoTEMPO (100 μM). Type I IFN expression level and extracellular HBV virion levels were measured. Data represent the mean ± SD of three independent experiments. *p < 0.05, **p < 0.01, and ***p < 0.001.

Previously, it was reported that mitochondrial stress-mediated oxidized DNA release into the cytosol contributed to IFN-I production *via* cGAS–STING axis (27, 28). Therefore, we investigated whether GV1001 exerts an anti-HBV effect *via* mitochondrial stress-mediated signaling. To this end, we first evaluated the mtROS and mtDNA levels in the cytoplasm. After the isolation of cytosolic mitochondrial DNA from cells, three regions of mtDNA—mtDNA 1, mtDNA 2, and mtDNA3, which detect ND5, ND1/ND2, and COII/ATPase6/8—were amplified. The GV1001 treatment significantly enhanced the cytosolic mtDNA levels in a dose-dependent manner (Figure 5A), suggesting that GV1001 enhances mtDNA release into the cytosol. To validate mtROS induction by GV1001, we evaluated mitochondrial superoxide *via* confocal microscopy and flow cytometry using MitoSOX. Our confocal images showed a dramatic increase in the mitochondrial fluorescence intensity of MitoSOX in HepG2-2.15 cells treated with 10 μM GV1001 compared with PBS-treated cells (Figure 5B and Supplementary Figure S1). The flow cytometry analysis also showed a significant histogram shift in HepG2-2.15 cells treated with 10 μM GV1001 compared with those treated with PBS at 6 and 12 h (Figure 5C and Supplementary Figure S2), suggesting that GV1001 enhanced mtROS production together with mtDNA.

Exposure to intracellular ROS in the mitochondria can cause oxidative mtDNA damage, indicated by increased mitochondrial 8-OHdG, which may thus be a useful biomarker to detect mtROS (29). We found that 8-OHdG, a ubiquitous marker of oxidative stress, was significantly elevated in a dose-dependent manner after 12 h of GV1001 treatment (Figure 5D).

Finally, we investigated the relationship between mitochondrial stress and the IFN-I-mediated anti-HBV effect by GV1001 using MitoTEMPO, a mitochondria-targeted antioxidant that protects mitochondria from oxidative damage. MitoTEMPO abrogated the anti-HBV effect and IFN-I production of GV1001 (Figure 5E). Taken together, our data suggest that GV1001 promotes mitochondrial stress-mediated oxidized mtDNA release into the cytosol, resulting in an IFN-I -mediated anti-HBV effect *via* the cGAS–STING axis.

### The Anti-HBV Effect of GV1001 Is Mediated in an Extracellular HSP90

**FIGURE 6:**
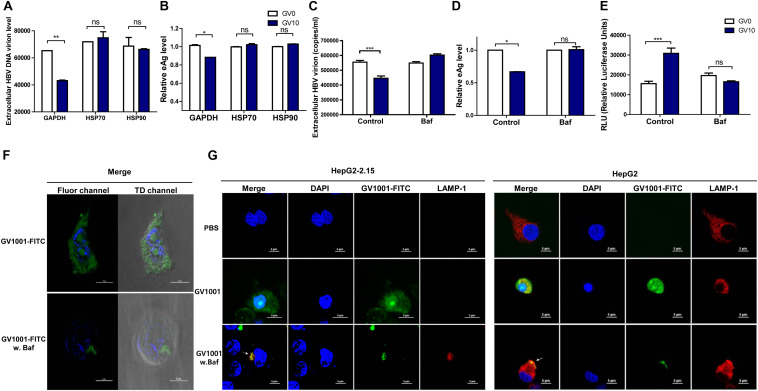
GV1001 exerts an anti-HBV effect *via* its eHSP-mediated cytosolic access and phagosomal escape. For confirmation of eHSP70- or eHSP90-dependent translocation of GV1001, HepG2-2.15 cells were pre-incubated with HSP70, HSP90, and GAPDH antibodies for 2 h. After treatment with GV1001 for 24 h, **(A,B)** extracellular HBV DNA and HBeAg were evaluated *via* qPCR and ELISA. **(C–E)** Extracellular HBV DNA, HBeAg, and type 1 IFN-dependent luciferase levels in HepG2-2.15 cells treated with GV1001 in the presence of Bafilomycin A1 (white, GV0; dark blue, GV10). **(F)** Confocal microscopic analysis showed the intracellular location of GV1001 with or without Bafilomycin A1 (100 nM) at 12 h. HepG2-2.15 cells stained with DAPI (blue) for nuclei and with FITC-labled GV1001 (GV1001-FITC, green) for the cytoplasmic display of GV1001. **(G)** The phagosome-associated protein LAMP-1 was detected in HepG2-2.15 and HepG2 cells with mouse anti-LAMP-1 and anti-mouse immunoglobulin-Alexa 594 (red). The white arrows represent the co-localization of GV1001-FITC with LAMP-1 (yellow). Data represent the mean ± SD of three independent experiments. *p < 0.05, **p < 0.01 and ***p < 0.001.

Previously, it was reported that GV1001 could translocate into the cell cytosol *via* extracellular HSP90 and HSP70 binding and exerted its antiviral effect on HCV or HIV-1 infections in eHSP90- and eHSP70-dependent manner (18). Therefore, we assessed the involvement of HSP90 and HSP70 in the anti-HBV effect of GV1001. Interestingly, the GV1001-mediated suppression of HBV replication in stable HepG2-2.15 cells was completely restored by anti-HSP90 or anti-HSP70 neutralizing antibody (Figures 6A,B). However, an isotype control GAPDH antibody showed no significant effect on GV1001 treatment. These results suggested that GV1001 regulates the anti-HBV effect through its interactions with eHSP90 and eHSP70.

### Cytosolic Localization of GV1001 After Phagosomal Escape Is Essential for the Antiviral Effect of GV1001 *via* IFN-I Signaling

In previous studies, GV1001, a cell-penetrating peptide (CPP), was localized in the cytoplasm rather than in the nucleus of MCF7, Huh-7, and HepG2 cells (30). In addition, it has been reported that the eHSP–CPP complex could be taken up by antigen-presenting cells and could escape into the cytosolic space from the phagosome (31). In this regard, drug delivery strategies for endolysosomal escape and cytosolic access mimicking the escape mechanism of pathogens (32) have emerged (33). Chitosan was reported to produce IFN-I in dendritic cells *via* its escape into the cytosol followed by endosome rupture, which can contribute to its strong adjuvant effect in vaccine application (34). Therefore, to investigate whether the anti-HBV effect of GV1001 is due to its CPP nature with cytosolic preferential localization, which could contribute to IFN-I production *via* vacuole escape, as shown in chitosan (35), we used Bafilomycin A, which inhibits the acidification of phagosomes. In the presence of Bafilomycin, treatment with GV1001 failed to reduce the extracellular HBV virion and HBeAg levels compared with those of the control group (Figures 6C,D). In addition, cells treated with GV1001 induced IFN-I expression, but no significant difference was shown in the Bafilomycin–GV1001 co-treated group (Figure 6E). We also observed differences between the cells treated with and without Bafilomycin in confocal images using GV1001-FITC. In a case without treatment, GV1001 was diffused or released throughout the cytosolic space, but when Bafilomycin was added, GV1001 was not diffused to the cytosolic space due to the inhibition of phagosome acidification (Figure 6F and Supplementary Figures S3A,B). In addition, to investigate the phagosomal escape and cytosolic access of GV1001, we observed the colocalization of GV1001 with the late endosomal/lysosomal marker LAMP-1 in the presence or absence of Bafilomycin A. In the presence of Bafilomycin, GV1001-FITC failed to escape into the cytosol and colocalized with LAMP-1 in both HepG2-2.15 and HepG2 cells, but without interruption of Bafilomycin A, the GV1001-FITC was diffused throughout the cytosolic space and did not colocalize with LAMP-1 (Figure 6G). Together, our results suggest that phagosome acidification or escape is required to induce IFN-I and the associated antiviral effect of GV1001.

### The Antiviral Effect of GV1001 Is Dependent on Enhanced HO-1 Expression

**FIGURE 7:**
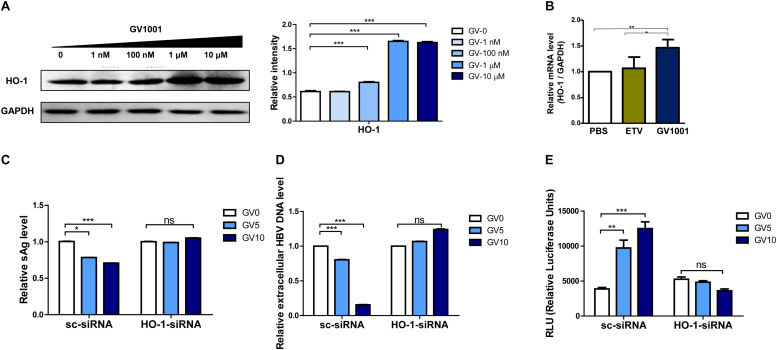
The anti-HBV effect of GV1001 is dependent on the enhanced HO-1 expression. **(A)** Western blots of 1.2x-WT plasmid-transfected HepG2 cells treated with GV1001 showing the HO-1 expression level. **(B)** Total RNA was extracted from HepG2-2.15 cells treated with phosphate-buffered saline, entecavir (30 nM), and GV1001 (10 μM) for 48 h, and RT-qPCR was conducted to determine the HO-1 mRNA levels. **(C–E)** HepG2 cells transfected with both 1.2x-WT plasmid and HO-1 or scramble siRNA were treated with GV1001. HBsAg **(C)**, extracellular HBV DNA **(D)**, and type 1 IFN luciferase levels **(E)** were measured. Data represent the mean ± SD of three independent experiments. *p < 0.05, **p < 0.01, and ***p < 0.001.

Heme-oxygenase-1 (HO-1) was shown to exert an anti-HBV effect *via* inhibition of the viral core/capsid formation (36). Next, we verified that GV1001 treatment increased HO-1 protein expression by Western blot analysis. We found that GV1001 enhanced HO-1 expression in a dose-dependent manner. Furthermore, GV1001 led to a reciprocal reduction of viral capsids in a dose-dependent manner, suggesting an inverse association between HO-1 expression and viral capsid formation (Figure 2C and Figure 7A). In addition, HO-1 gene transcription was significantly increased in GV1001-treated HepG2 cells (Figure 7B). Therefore, to determine the HO-1 involvement in viral capsid formation, we compared the anti-HBV effect of GV1001 between HBV genome-transfected HepG2 cells cotransfected with HO-1 siRNA or scramble siRNA. Notably, the reduced HBsAg, extracellular HBV virion levels, and increased IFN production level mediated by GV1001 in scramble siRNA-transfected HepG2 cells were not shown in HO-1 siRNA-transfected cells (Figures 7C–E). These results suggested that the anti-HBV effect of GV1001 is dependent on enahnced HO-1 expression.

## Discussion

Current NA-based antiviral agents can control, but not completely cure, HBV infection in chronic patients due to the persistence of HBV cccDNA in infected hepatocytes (37). Exogenous IFN-α treatment for CHB patients can lead to complete viral clearance in a proportion of patients. However, this treatment was not efficacious in genotype C-infected patients, and high doses are not tolerated (38). Therefore, the development of new drugs for the efficient elimination of HBV cccDNA is urgently needed. A novel candidate anti-HBV drug, GV1001, a telomerase-derived peptide whose antiviral effects against HCV and HIV-1 have already been described, was introduced in this study (17, 18). Here we demonstrated that the anti-HBV effect of GV1001 is due to its capacity to produce endogenous IFN-I *via* mitochondrial DNA stress. We also demonstrated that mtDNA stress elicited by GV1001 is attributed to cell cytosol access *via* phagosomal escape after eHSP-mediated cell penetration, which leads to the release of oxidized mtDNA into the cytosol. The resulting cytosolic mtDNA following GV1001 treatment induced antiviral innate immune responses *via* IFN-I production in a STING-dependent manner (Graphical Abstract).

Hepatitis B virus is a stealth virus capable of escaping IFN-I-dependent antiviral responses (39). HBV has developed various strategies to escape IFN-I production through RNA sensing *via* RIG-I or TLR-3 or -7 pathogen recognition receptors, which could be mediated by virally encoded proteins, including HBsAg, HBeAg, HBV Pol, or HBxAg (40–43). Recently, an HBV strategy to modulate distinct STING pathways for IFN-I production has been introduced (44).

However, our *in vitro* and *in vivo* studies proved that mtDNA stress-mediated IFN-I production *via* the STING-IRF3 axis by GV1001 could override the IFN-I escape mechanism (44), identifying GV1001 as an HBV treatment. Furthermore, cytosolic mtDNA following GV1001 treatment can induce the production of other antiviral cytokines, such as IL-1ß, *via* NLRP inflammasome activation as well as IFN-I production in a cGAS–STING-dependent manner (45), possibly from liver Kupffer cells, providing an additional benefit of GV1001 over exogenous IFN treatment.

Previously, we identified GV1001 as a CPP, which preferentially localized into the cell cytosol in an eHSP-dependent manner, suggesting potential uses for cell delivery of various pharmaceutical agents, such as proteins, DNA, or siRNA (30). Here, our data clearly demonstrated that blocking the eHSP-mediated cell entry of GV1001 led to complete inhibition of antiviral activity (Figures 6A,B). Furthermore, we found that treatment with Bafilomycin, an inhibitor of phagosome acidification, inhibited the antiviral and IFN-I effects of GV1001 (Figures 6C–E), suggesting that the antiviral activity of GV1001 depends on phagosomal escape after eHSP-mediated cell entry. In fact, some pathogens, such as *Mycobacterium tuberculosis* (46), *Mycobacterium abscessus* (32), or *Listeria monocytogenes* (47), were shown to exploit host innate immune systems *via* enhanced IFN-I production by mitochondrial stress after active phagosome rupture, promoting their virulence. This finding suggests that the antiviral mechanism of GV1001 may mimic the strategy of some pathogens to modulate the host innate immune response, as shown in the mechanism of chitosan as a vaccine adjuvant (48).

Previously, HO-1 was shown to exert an anti-HBV effect at a post-transcriptional stage by decreasing the stability of HBV capsid formation and blocking the refilling of nuclear HBV cccDNA (36). Furthermore, a byproduct of HO-1, biliverdin, could reduce HCV replication by increasing IFN-I signaling (49). Our data also indicated that GV1001 inhibited viral capsid formation in a dose-dependent manner *via* enhanced HO-1 expression (Figure 7A). Furthermore, the HO-1 knockdown experiment demonstrated that siRNA-mediated HO-1 inhibition abrogated the IFN-I production and the anti-HBV effects induced by GV1001, suggesting that GV1001 could exert an anti-HBV effect *via* inhibiting the virion capsid formation through the HO-1–IFN-I axis (Figures 7C–E).

In addition, our data, showing that GV1001 enhances IFN-I production, suggest that GV1001 could play dual roles in an anticancer vaccine: as a cancer-associated telomerase antigen and as an adjuvant *via* IFN-I-mediated anticancer cell-mediated immune responses. This finding could explain why GV1001 shows the strongest anticancer vaccine effect of various telomerase-derived peptides.

In summary, our data indicated that the cell-penetrating and cytosolic localization capacity of GV1001 exerts antiviral effects in HBV infections *via* mitochondrial stress-mediated IFN-I production (Graphical Abstract). These results suggest the potential use of GV1001, a peptide proven to be safe for human use, as an anti-HBV drug, which can be synergistically used with NA drugs.

## Data Availability Statement

All datasets generated for this study are included in the article/Supplementary Material.

## Ethics Statement

The animal study was reviewed and approved by the Institutional Animal Care and Use Committee (IACUC) of the Seoul National University College of Medicine.

## Author Contributions

Y-MC, HK, and B-JK contributed to the conception and the design of the study and performed the statistical analysis. Y-MC, HK, S-AL, and S-YL organized the database. Y-MC, HK, and S-AL performed the lab work. Y-MC and B-JK wrote the draft of the manuscript and revised the manuscript. All the authors read and approved the submitted version.

## Conflict of Interest

The authors declare that the research was conducted in the absence of any commercial or financial relationships that could be construed as a potential conflict of interest.

## Abbreviations

cccDNA: covalently closed DNA
CHB: chronic hepatitis B
HBeAg: hepatitis B e-antigen
HBsAg: hepatitis B surface antigen
HBV: hepatitis B virus
HO-1: heme oxygenase-1
LAM: lamivudine
mtROS: mitochondrial ROS
NAs: nucleos(t)ide analogs
pgRNA: pregenomic RNA
qPCR: quantitative polymerase chain reaction
RT-qPCR: reverse transcription-quantitative polymerase chain reaction
type 1 IFN: type 1 interferon.
