# Supplementary material for: A Telomerase-Derived Peptide Exerts an Anti-Hepatitis B Virus Effect via Mitochondrial DNA Stress-Dependent Type I Interferon Production
Source: Front Immunol. 2020 May 21;11:652. doi: 10.3389/fimmu.2020.00652 (PMC7253625; doi:10.3389/fimmu.2020.00652)
Supplement: Supplementary file 1 [file Data_Sheet_1.PDF]

SUPPLEMENTARY MATERIAL

Supplementary figures and figure captions

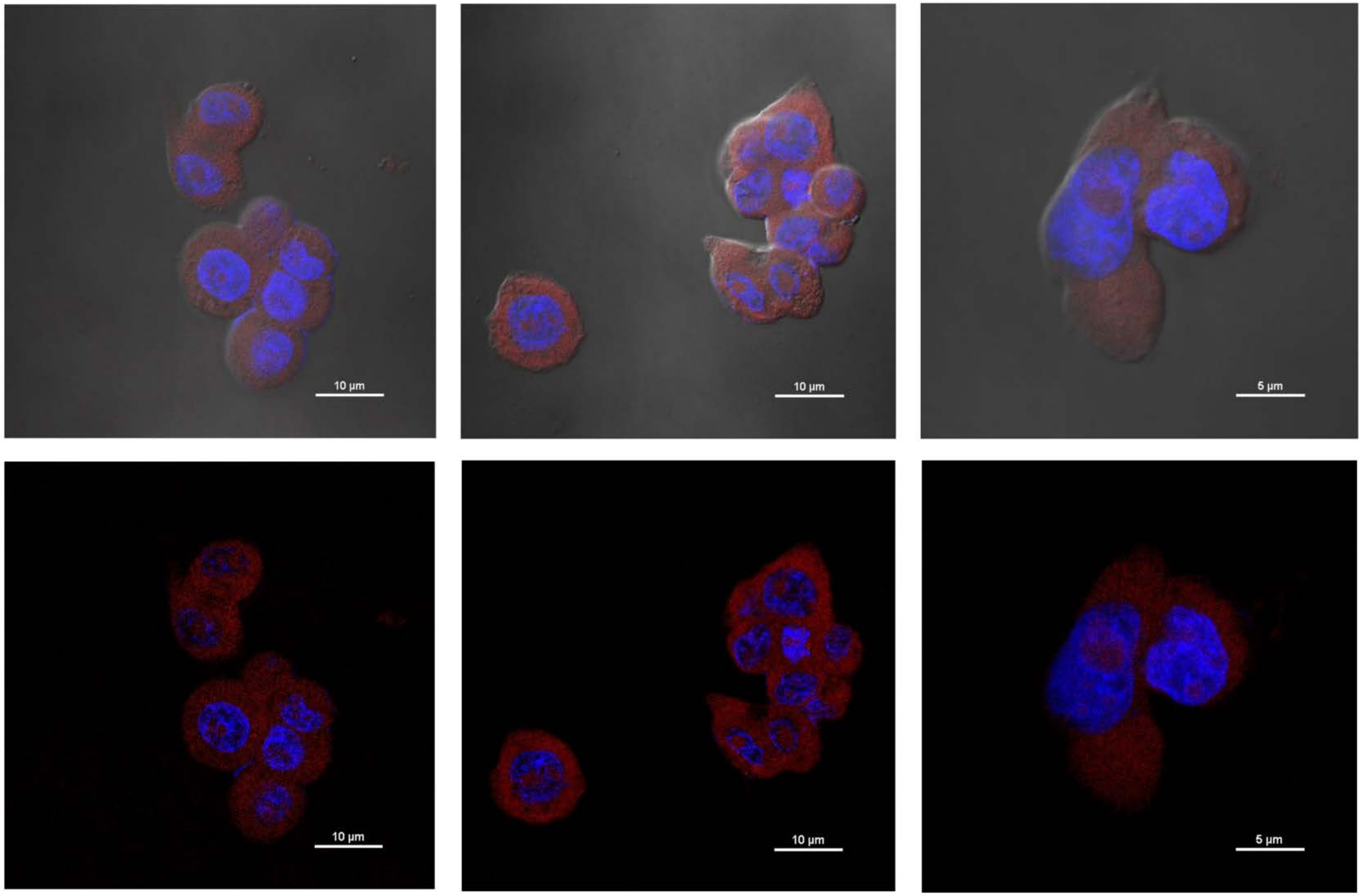

Supplementary Figure 1. GV1001 induced cytoplasmic mtROS.
Confocal images of HepG2-2.15 cells showing elevated mitochondrial superoxide levels following treatment with 10 μM GV1001 for 12 h.

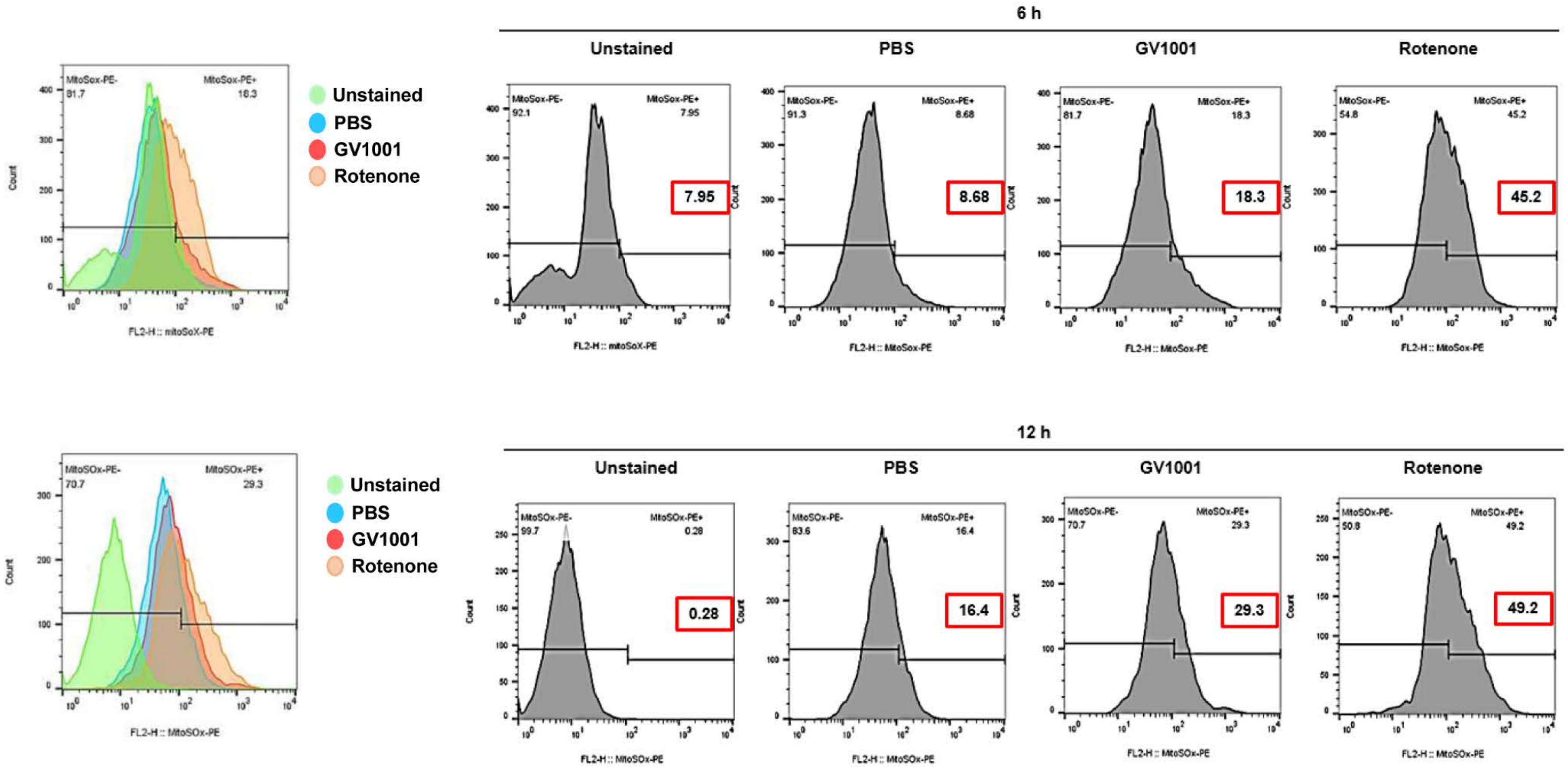

Supplementary Figure 2. Cytoplasmic mtROS is necessary for GV1001 to drive Type 1 IFN.
Representative histograms of flow cytometric assays showing shifted histograms following treatment with GV1001 for 6 h and 12 h.

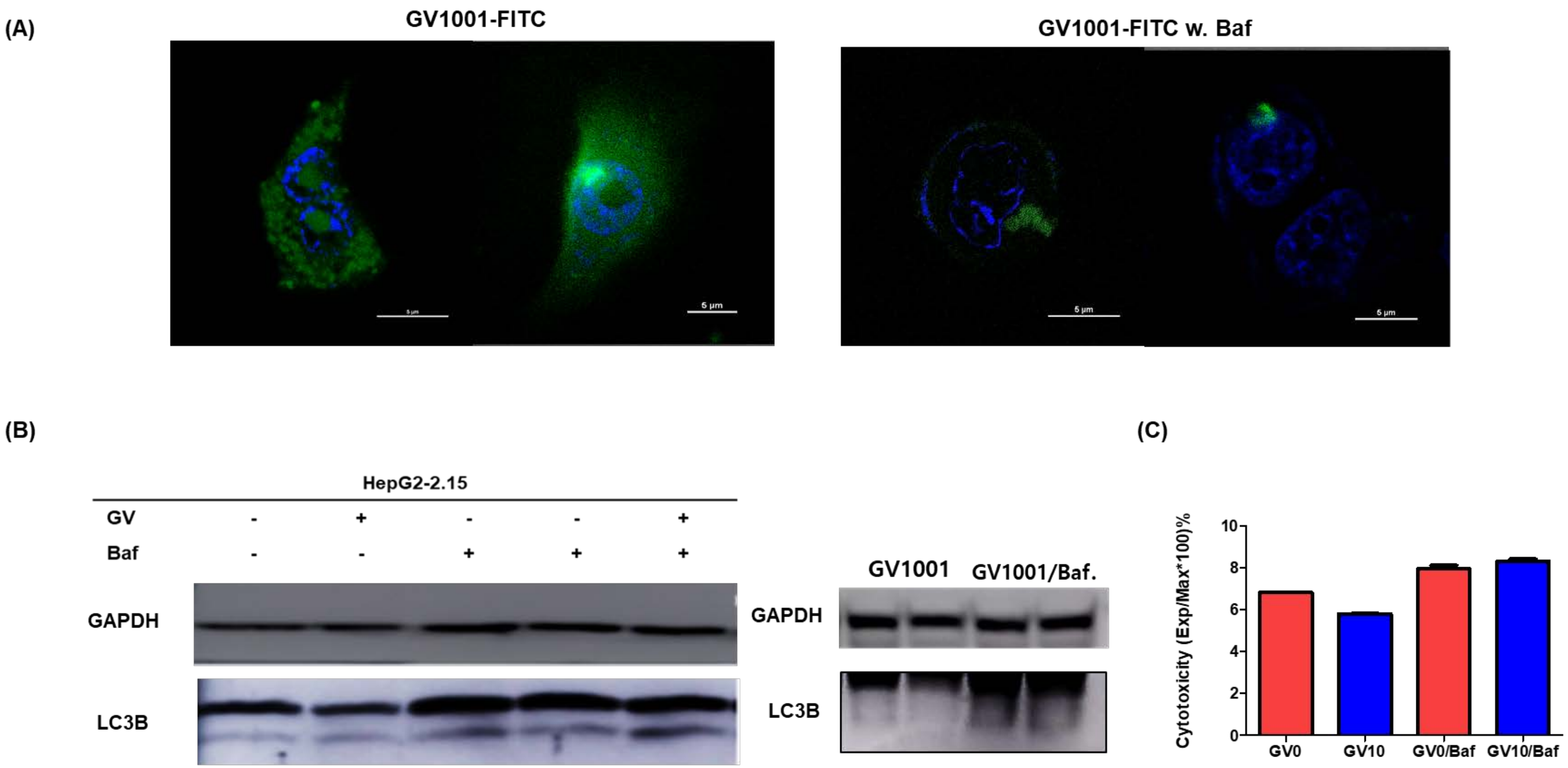

Supplementary Figure 3. Lysosomal rupture is essential for the antiviral effect of GV1001

(A) Confocal microscopic analysis showed the intracellular location of GV1001 with or without Bafilomycin A1 at 4 h.

(B) LC3B Western blots of HepG2-2.15 cells treated with or without GV1001 in the presence of Bafilomycin. Bafilomycin inhibited protein degradation and led to accumulation of LC3-II.

(C) A lactate dehydrogenase (LDH) assay was carried out on HepG2-2.15 cells to determine the cytotoxicity induced by GV1001 and Bafilomycin A1.
